# High-Throughput Calculations on the Decomposition Reactions of Off-Stoichiometry GeSbTe Alloys for Embedded Memories

**DOI:** 10.3390/nano11092382

**Published:** 2021-09-13

**Authors:** Omar Abou El Kheir, Marco Bernasconi

**Affiliations:** Dipartimento di Scienza dei Materiali, Università di Milano-Bicocca, Via R. Cozzi 55, I-20125 Milano, Italy; o.abouelkheir@campus.unimib.it

**Keywords:** phase change materials, embedded electronic memories, Density Functional Theory, high-throughput calculations

## Abstract

Chalcogenide GeSbTe (GST) alloys are exploited as phase change materials in a variety of applications ranging from electronic non-volatile memories to neuromorphic and photonic devices. In most applications, the prototypical Ge${}_{2}$Sb${}_{2}$Te${}_{5}$ compound along the GeTe-Sb${}_{2}$Te${}_{3}$ pseudobinary line is used. Ge-rich GST alloys, off the pseudobinary tie-line with a crystallization temperature higher than that of Ge${}_{2}$Sb${}_{2}$Te${}_{5}$, are currently explored for embedded phase-change memories of interest for automotive applications. During crystallization, Ge-rich GST alloys undergo a phase separation into pure Ge and less Ge-rich alloys. The detailed mechanisms underlying this transformation are, however, largely unknown. In this work, we performed high-throughput calculations based on Density Functional Theory (DFT) to uncover the most favorable decomposition pathways of Ge-rich GST alloys. The knowledge of the DFT formation energy of all GST alloys in the central part of the Ge-Sb-Te ternary phase diagram allowed us to identify the cubic crystalline phases that are more likely to form during the crystallization of a generic GST alloy. This scheme is exemplified by drawing a decomposition map for alloys on the Ge-Ge${}_{1}$Sb${}_{2}$Te${}_{4}$ tie-line. A map of decomposition propensity is also constructed, which suggests a possible strategy to minimize phase separation by still keeping a high crystallization temperature.

## 1. Introduction

GeSbTe (GST) phase change alloys have been deeply investigated over the last two decades for a wide range of applications ranging from non-volatile electronic memories (phase change memories, PCMs) [1,2,3,4] to neuromorphic computing [5,6], optical disks [7] and several other optical devices [8,9]. These applications rest on two peculiar properties of GST alloys: a reversible and very rapid transition between the amorphous and crystalline phases upon heating and a large contrast in the electronic and optical properties between the two phases, which allows the encoding of the digital information.

In PCMs, the phase transition is induced by Joule heating. During reset, the crystalline phase is rapidly brought above the melting temperature by intense and short current pulses and the amorphous phase is then recovered when the bias is removed due to fast heat dissipation in the surrounding materials. During set, longer and less intense current pulses at a voltage above the Ovonic threshold [10] raise the temperature in between the glass transition temperature $T_{g}$ and the melting temperature to induce the recrystallization of the amorphous material [1,3,11,12]. Phase change materials are bad glass formers and rapidly crystallize above *T*${}_{g}$, which is often identified with the crystallization temperature $T_{X}$ [13].

The prototypical phase change compound used in PCMs is the Ge${}_{2}$Sb${}_{2}$Te${}_{5}$ (GST225) alloy which can be seen as a pseudobinary compound lying on the GeTe-Sb${}_{2}$Te${}_{3}$ tie-line. In memory operation, the amorphous phase of GST225 crystallizes into a metastable rocksalt phase and not in the thermodynamically stable trigonal phase due to kinetic hindrances. Note that in the following we will indicate with GSTXYZ the alloy with composition Ge${}_{x}$Sb${}_{y}$Te${}_{z}$. The amorphous phase of GST alloys is obviously metastable below $T_{X}$ and it is subject to aging with the increase of the electrical resistance with time [14]. Recrystallization below $T_{X}$ is also possible on a longer time scale due to the stochasticity of the crystal nucleation process, which leads to data loss of the memory [3]. Data retention thus requires minimizing these unwanted events by suitably tuning the activation energy for the nucleation process at temperatures below *T*${}_{g}$.

Although PCMs based on GST alloys are at an advanced stage of development and have reached the global market [15], the exploration of materials in this class is still extensively pursued to enlarge their field of applicability. For instance, GST alloys with $T_{X}$ higher than that of GST225 are needed for application in embedded memories of interest for the automotive sector [16]. To this end, Ge-rich GST alloys are emerging as promising materials with $T_{X}$ raising up to 570 K by increasing the Ge content along the Ge-Sb${}_{2}$Te${}_{3}$ pseudobinary line [17,18] as opposed to a value $T_{X}$ in the range 420–440 K for GST225 [19]. Ge-rich alloys along the Ge-Ge${}_{2}$Sb${}_{2}$Te${}_{5}$ pseudobinary line [20] or on the Sb-GeTe isoelectronic line (the same average number of p electrons per atom) [21] have also been explored to improve data retention. The high $T_{X}$ of Ge-rich alloys has been ascribed to the slow-down of the crystallization kinetics due to the mass transport involved in the phase separation into crystalline Ge and less Ge-rich crystalline alloys [22]. In fact, the segregation phenomena require long length diffusion of the atomic species, which would imply a longer incubation time for the formation of supercritical crystalline nuclei. This process was reported in the crystallization of the as-deposited amorphous films and during forming (initialization) of the memory cells [17,18,22,23,24,25,26]. The presence of a reversible phase separation during set/reset is instead less clear. The inhomogeneity of the active material resulting from phase separation has, however, some drawbacks such as a resistance drift in the set (crystalline) state as well, a cell-to-cell variability and possibly reduced endurance [16]. There is therefore room for improvement in the choice of the best composition of the alloy to minimize these detrimental effects. To this end, better knowledge of the decomposition process than is actually available is mandatory. The decomposition process is highly non trivial because, during the operation of the memory, the amorphous phase is supposed to crystallize into metastable cubic phases due to kinetic hindrances, as occurs for GST225, and not necessarily into the thermodynamically stable compounds, which encompass only the unary elements and the pseudobinary compounds along the GeTe-Sb${}_{2}$Te${}_{3}$ and Sb-Sb${}_{2}$Te${}_{3}$ tie-lines (including the end points) on the Ge-Sb-Te ternary phase diagram.

Atomistic simulations can provide useful information in this respect. In a very recent work, for instance, molecular dynamics simulations based on Density Functional Theory (DFT) were applied to study the amorphous phase of Ge-rich GST along the Ge-GST124 pseudobinary line [27]. The simulations revealed that the amorphous phase is stable with respect to phase separation into amorphous Ge and amorphous GST124 only for Ge content below or equal to 50 atomic %. Phase separation during crystallization was, however, not addressed in Ref. [27].

In this work, we fill this gap by studying the decomposition process during the crystallization of GST alloys by means of high-throughput DFT calculations. In particular, we have constructed the convex hull from the calculations of the DFT total energy of the stable compounds. For all GST cubic alloys in the central part of the ternary phase diagram, we then obtained the distance of their free energy from the convex hull which gives a measure of the metastability of the alloy. These data allowed us to compute the reaction free energy for the decomposition of Ge-rich GST alloys into the crystalline Ge (and eventually Sb and Te), a less Ge-rich alloy, and the GeTe and Sb${}_{2}$Te${}_{3}$ binary compounds. The calculations provide a map for the decomposition paths of Ge-rich alloys of any composition and allow us to quantify the propensity to decompose of any alloy and its most probable decomposition products. This approach is exemplified by studying the decomposition pathways of Ge-rich alloys on the Ge-GST124 pseudobinary line. It turns out that several decomposition channels compete, which can lead to the formation of a nanocomposite of crystalline grains with different compositions.

## 2. Computational Details

We performed DFT calculations by employing the exchange and correlation functional due to Perdew, Burke and Ernzerhof (PBE) [28] and the norm-conserving pseudopotentials by Goedeker, Teter and Hutter [29,30] within the Quickstep method as implemented in the CP2k package [31]. The Kohn–Sham orbitals were expanded in Gaussian-type orbitals of a valence triple-zeta-valence plus polarization basis set while the electronic density was expanded in a basis set of plane waves up to a kinetic energy cutoff of 100 Ry. We also included the van der Waals (vdW) interactions, which have been shown to affect the structural properties of GST compounds [32,33], by including the semiempirical correction due to Grimme [34]. We modeled all GSTXYZ alloys in the cubic rocksalt phase for compositions in the central part of the Ge-Sb-Te ternary phase diagram in which a single element (Ge, Sb or Te) in the alloy does not exceed 60 atomic %. This choice is motivated by the fact that for higher Ge, Sb or Te content we might expect to have either defected tetrahedral geometry, the trigonal layered structures of Sb or more complex structures for very high Te fraction. Since we are interested in the possible switching between the amorphous phase and a cubic crystalline phase, this restriction to the central part of the ternary phase diagram is well justified.

The metastable cubic phase for all compositions was modeled in 216-atom supercells in the rocksalt geometry analogous to the metastable cubic phase of GST225. In cubic GST225, Sb and Ge atoms occupy randomly the cation sublattice with 20% of stoichiometric vacancies, while Te atoms fully occupy the anionic sublattice. Stoichiometric vacancies in GST225 ensure the presence of exactly three p electrons per site on average, which leads to a closed shell system [35].

For GSTXYZ alloys with an atomic fraction of Ge larger than 50%, the cationic sublattice is occupied by Ge only and the anionic sublattice is occupied randomly by Sb, Ge and Te. For Sb-rich alloys, antimony is supposed to behave as an amphoteric element by occupying both the anionic and cationic sublattices. For compositions with more than three p electrons per site, we included vacancies in the cationic sublattice to enforce the condition of three p electrons per site on average. To properly include disorder on the cationic or on both the cationic and anionic sublattices, three different models for each composition were generated by using special quasi-random structures (SQS) [36] with the package of Ref. [37]. The ordered compounds Ge, Te ($P3_{1}21$ space group) [38], Sb ($R\bar{3}m$ space group) [39], GeTe (R3m space group) [40] and Sb${}_{2}$Te${}_{3}$ ($R\bar{3}m$ space group) [41] were modeled with 216, 192, 216, 162 and 270 atom supercells. The atomic positions and cell edges of all models were fully optimized with a convergence threshold of 5 mRy/$a_{o}$ for forces and a pressure tolerance of 0.01 GPa. The cell of the metastable phases was first optimized by keeping the cubic symmetry of the supercell, and subsequently also the cell angles were allowed to change. Cell optimizations were performed by restricting the Brillouin zone (BZ) integration to the supercell Γ point. The total energy of the models was then computed with a 3 × 3 × 3 k-point mesh in the supercell BZ. In order to estimate the reaction free energies, we also included the configurational entropy in the crystalline phases due to disorder as given by $S=\frac{-k_{B}}{2}\Sigma_{i,j}x_{i,j}$ln$\left(x_{i,j}\right)$, where the *j* index runs over the sublattices (i.e., cationic and anionic) and the *i* index runs over the atomic species which occupy a particular sublattice, *k*${}_{B}$ is the Boltzmann constant and *x*${}_{i,j}$ is the molar fraction. In the following, we refer to the (reaction) free energy, which includes the total energy at zero temperature and the configurational free energy at 300 K. Calculations on selected possible decomposition pathways have shown that the phononic contribution to the reaction free energy is very small (a few meV/atom) [42] and it has thus been omitted in the data reported hereafter.

## 3. Results

**Figure 1:**
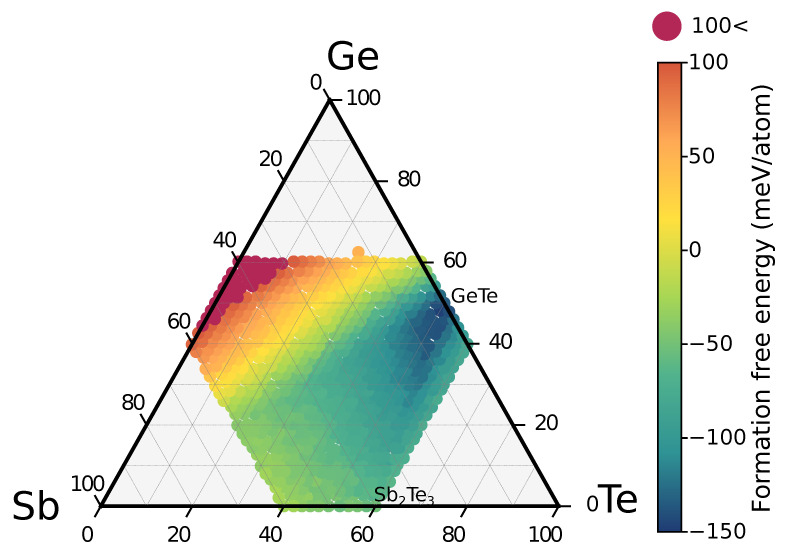
Map of the formation free energy of GST alloys in the metastable cubic phase (at 300 K, see text). The GeTe compound is instead in the stable trigonal phase. The topmost point corresponds to GST512.

We first computed the formation free energy of GSTXYZ alloys with respect to the Sb, Ge and Te elements in their standard state. The map of the formation free energy for cubic alloys in the ternary phase diagram is shown in Figure 1. Each point on the map corresponds to a different composition for a total number of 698 alloys uniformly spaced in the central part of the phase diagram. We remind that the free energy is obtained from the total energy at zero temperature plus the configurational free energy at 300 K due to disorder averaged over three SQS models for each composition (see Section 2). A positive formation free energy means that the alloy is unstable with respect to phase separation into the elements Sb, Ge and Te. This is the case for Sb-rich alloys on the left part of the phase diagram. GeSb itself does not exist as a thermodynamically stable compound although a metastable, tetragonally distorted rocksalt phase was reported [43]. The formation free energy is lower (more negative) for alloys along the GeTe-Sb${}_{2}$Te${}_{3}$ tie-line and on the Sb-GeTe isoelectronic line. Note that alloys around Sb${}_{2}$Te${}_{3}$ are all in the cubic phase as well. A metastable rocksalt phase of Sb${}_{2}$Te${}_{3}$ with vacancies on 1/3 of the cationic sites was also recently found experimentally [44].

The energy of the unary systems and of the trigonal binary compounds GeTe and Sb${}_{2}$Te${}_{3}$ is used to construct the convex hull, which, for a ternary system, is the two dimensional surface formed by the ordered compounds that have an energy lower than that of any other structure or any linear combination of structures that provide the proper composition. Compositions at the vertices of the convex hull are thus thermodynamically stable compounds while the tie-lines connecting two compounds are the edges of the convex hull. All other structures have an energy that falls above the set of tie-lines [45,46,47]. The convex hull represents the Gibbs free energy of the alloy at zero temperature.

**Figure 2:**
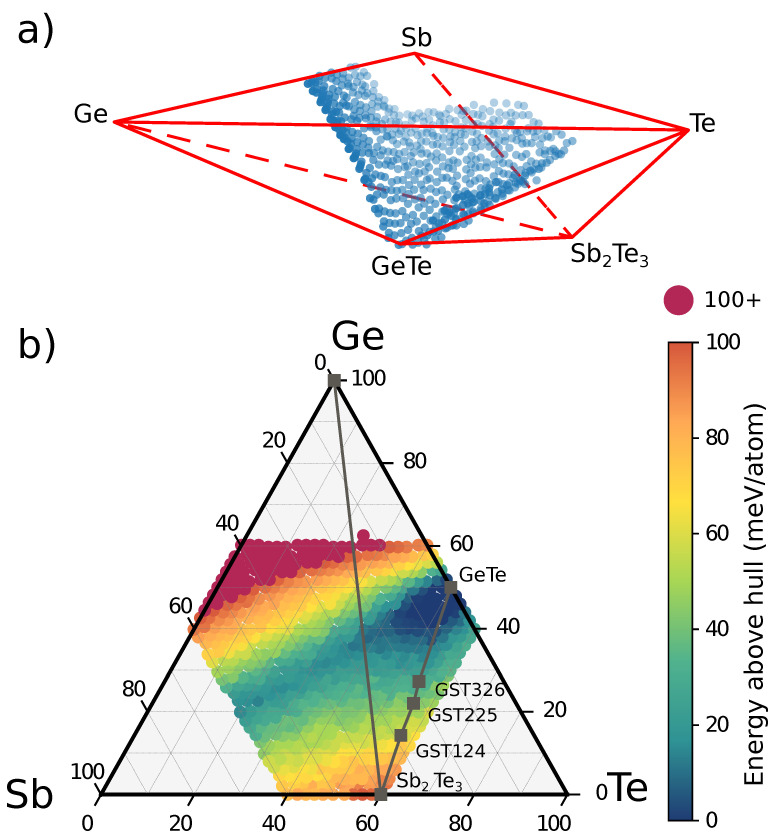
(**a**) Convex hull in the Ge-Sb-Te phase diagram. The energy of the elements in their standard state is set to zero. The figure shows a simplified convex hull in which the stable pseudobinary GeTe-Sb${}_{2}$Te${}_{3}$ compounds and the stable Sb${}_{n}$Te compounds have been brought on the GeTe-Sb${}_{2}$Te${}_{3}$ and Sb-Sb${}_{2}$Te${}_{3}$ tie-lines (see text). The formation free energy of the different alloys is also shown by blue points only for compositions with a negative formation free energy (see Figure 1). (**b**) Map of the distance of the formation free energy (meV/atom) of the cubic alloys from the convex hull. The shorter the distance the higher is the degree of metastability of the alloy. The GeTe-Sb${}_{2}$Te${}_{3}$ and Ge-Sb${}_{2}$Te${}_{3}$ tie-lines are also shown in panel (**b**) along with the points corresponding to some stable (GeTe)${}_{n}$(Sb${}_{2}$Te${}_{3}$)${}_{m}$ pseudobinary compounds.

Actually, several pseudobinary (GeTe)${}_{n}$(Sb${}_{2}$Te${}_{3}$)${}_{m}$ alloys [48,49,50] and Sb${}_{n}$Te compounds [51] are known to be thermodynamically stable and they thus represent vertices of the convex hull. However, the energies of (GeTe)${}_{n}$(Sb${}_{2}$Te${}_{3}$)${}_{m}$ compounds, such as GST147, GST124, GST225, GST326, GST528 and so forth, are very close to the (GeTe)-(Sb${}_{2}$Te${}_{3}$) edge of the convex hull. The same is true for the Sb${}_{n}$Te compounds along the Sb-Sb${}_{2}$Te${}_{3}$ tie-line [51]. Therefore, we considered a simplified convex hull including only Ge, Sb, Te and the binary GeTe and Sb${}_{2}$Te${}_{3}$ trigonal compounds, which was built by using the qhull code [52]. The geometry of the convex hull with the free energy points of the different cubic alloys is sketched in Figure 2a. A map of the distance of the free energy of the different cubic alloys from the hull is shown instead in Figure 2b. The shorter the distance, the higher the degree of the metastability of the alloy.

Note that the alloys on the Sb-GeTe isoelectronic line are particularly stable since their energy is very close to that of the convex hull. In fact, the GST212 alloy on the Sb-GeTe line was indicated a few years ago as the starting point to obtain, by Ge enrichment, a “golden composition” for embedded memories [21]. The high metastability (low distance from the convex hull) corresponds to a low propensity to decompose as we will discuss later on. Although cubic alloys along the GeTe-Sb${}_{2}$Te${}_{3}$ tie line have a large and negative formation energy (see Figure 1) their distance from the convex hull is larger than that of alloys on the Sb-GeTe line. In fact, the edge of the convex hull along the GeTe-Sb${}_{2}$Te${}_{3}$ tie-line is very close to the energy of the trigonal phases which means that the cubic phases are about 50–60 meV/atom higher in energy than the trigonal ones (see also the very recent DFT work of Ref. [53]).

The map of the distances from the convex hull allows us to study the possible decomposition pathways of any alloy in the phase diagram as follows. We consider the decomposition free energy of the GSTXYZ cubic alloy according to the reaction:

(1) $$GSTXYZ\to a\;GSTX^{\prime }Y^{\prime }Z^{\prime }+b\;Sb\;+\;c\;Ge\;+\;d\;Te\;+\;e\;Sb_{2}Te_{3}\;+\;f\;GeTe.$$

The GSTX${}^{\prime }$Y${}^{\prime }$Z${}^{\prime }$ alloy and the Sb${}_{2}$Te${}_{3}$ compound are in the cubic phase as well, while the other unary systems and the binary GeTe compound are in their ground states (zero temperature). For a given ternary reactant and product the decomposition path is assigned by first maximizing the fraction (*a*) of the ternary product and then the fraction (*e* or *f*) of the binary products. These two constraints uniquely assign the fraction of the unary products. For each GSTXYZ alloy we can then construct a map of the decomposition free energy into a generic GSTX${}^{\prime }$Y${}^{\prime }$Z${}^{\prime }$ cubic alloy in the ternary phase diagram. This map highlights which decomposition paths are more likely to be seen during the crystallization of the amorphous phase. Note that the energy of the amorphous phase is always higher than the energy of the cubic phase at the same composition, as we verified with DFT calculations for selected compositions whose amorphous 216-atom models were generated by quenching from the melt following the same protocol we used in previous works [54,55,56,57].

As an example of this methodology, we have computed the decomposition map of the three alloys, GST312, GST412 and GST512, on the Ge-GST124 pseudobinary line, whose amorphous phase was investigated by DFT simulations in Ref. [27].

**Figure 3:**
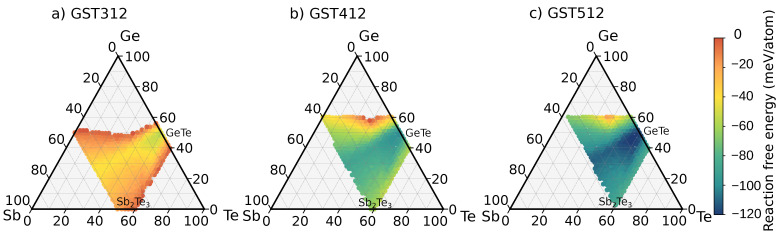
Maps of the decomposition pathways during crystallization of (**a**) GST312, (**b**) GST412, and (**c**) GST512. The color code gives the reaction free energy (meV/atom) to form the cubic alloys on the ternary phase diagram starting from the reactant, i.e., GST312 in panel (**a**) (see text).

The decomposition maps for the three alloys are compared in Figure 3. A point for the generic GSTX${}^{\prime }$Y${}^{\prime }$Z${}^{\prime }$ alloy in the decomposition map of GST312, for instance, gives the value of the decomposition free energy for the formation of GSTX${}^{\prime }$Y${}^{\prime }$Z${}^{\prime }$ according to Equation (1). For example, the reaction free energy for the formation of GST334 from GST312 corresponds to the reaction GST312 → 1/3 GST334 + 2/3 GeTe + 4/3 Ge. Since the alloys are modeled by a 216-atom supercell, the actual composition is not exactly given by the reaction above but by Ge${}_{107}$Sb${}_{36}$Te${}_{73}$→$\frac{36}{62}$ Ge${}_{64}$Sb${}_{62}$Te${}_{84}$+ $\frac{751}{31}$ GeTe+$\frac{1414}{31}$ Ge, due to the constraints imposed by the finite size of the supercell.

A negative reaction free energy indicates an exothermic reaction. The larger and more negative the reaction free energy is, the more favored is the corresponding decomposition pathway.

In the maps shown in Figure 3, we report only exothermic reactions which form GSTX${}^{\prime }$Y${}^{\prime }$Z${}^{\prime }$ in an amount that corresponds at least to 33 atomic % (1/3) of the reactant. Only under these conditions do we consider GSTX${}^{\prime }$Y${}^{\prime }$Z${}^{\prime }$ the main product of the decomposition pathway and show a point on the decomposition map. The composition of the reactant GSTXYZ obviously sets constraints on the possible products, irrespective of the reaction free energy. On the other end, the reaction free energy becomes larger and more negative for a wider part of the phase diagram (the products) by increasing the fraction of Ge (compares GST312 with GST512 in Figure 3). Note that most exothermic reactions lead to the formation of alloys on the Sb-GeTe and GeTe-Sb${}_{2}$Te${}_{3}$ lines with a larger weight for alloys close to GeTe.

**Figure 4:**
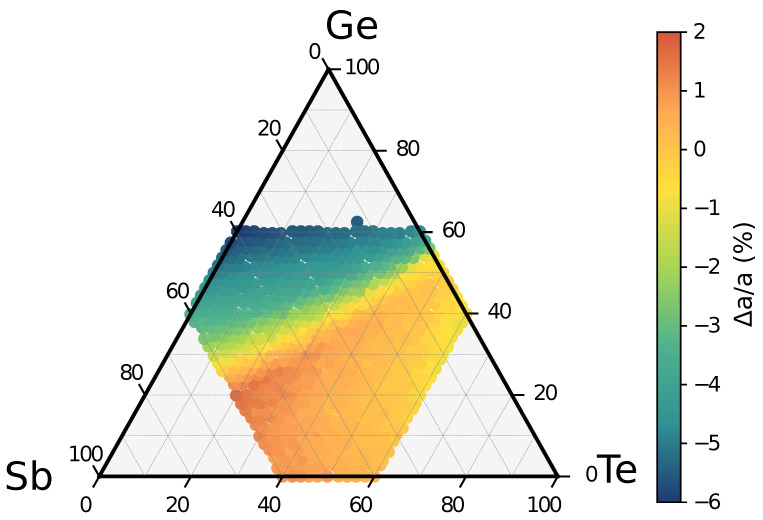
Map of the lattice parameter of the cubic phase of GST alloys in the ternary phase diagram. The lattice parameter is shown as the relative difference $\left(a-a_{225}\right)/a_{225}$ in percentage (%) with respect to the lattice parameter of GST225 ($a_{225}$ = 6.0293 Å) [49].

It is clear that there are several competitive channels for decomposition with very similar reaction free energies for all the three alloys considered in Figure 3 (GST312, GST412, and GST512). The GSTX${}^{\prime }$Y${}^{\prime }$Z${}^{\prime }$ products of these reactions can hardly be discriminated by X-ray diffraction data. The lattice parameter is in fact very similar for the majority of the alloys in the cubic phase as shown in Figure 4, which reports a map of the deviation of the equilibrium lattice parameter from that of GST225 for the different cubic alloys in the ternary phase diagram.

**Figure A1:**
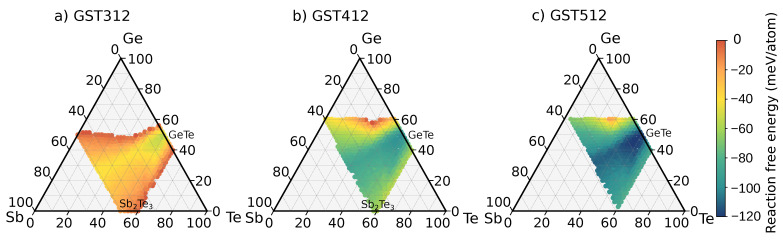
Maps of the decomposition pathways during crystallization of (**a**) GST312, (**b**) GST412, and (**c**) GST512. The color code gives the reaction free energy (meV/atom) to form the cubic alloys on the ternary phase diagram starting from the reactant, i.e., GST312 in panel (**a**) (see text). In these maps the Sb${}_{2}$Te${}_{3}$ product is considered in the trigonal phase as opposed to the maps in Figure 3 where the cubic phase was used for Sb${}_{2}$Te${}_{3}$ as well.

We thus expect the coexistence of different cubic alloys resulting from the crystallization of the amorphous phase of Ge-rich GST alloys during the operation of the memory, and possibly also a cell-to-cell variability due to the presence of several competitive channels for decomposition. The decomposition propensity is, however, different for the three alloys on the Ge-GST124 pseudobinary line investigated here. The alloy that is richer in Ge is in fact more prone to decompose into crystalline Ge and a less Ge-rich alloy, as shown by the presence of a larger blue (more exothermic reactions) region in the decomposition map of GST512. We also computed the decomposition maps of the three alloys by considering both GeTe and Sb${}_{2}$Te${}_{3}$ in the trigonal phase. These new maps are shown in Figure A1 in the Appendix A; they are nearly indistinguishable from those of Figure 3 because for Ge-rich alloys there are very few decomposition channels leading to crystalline Sb${}_{2}$Te${}_{3}$.

**Figure 5:**
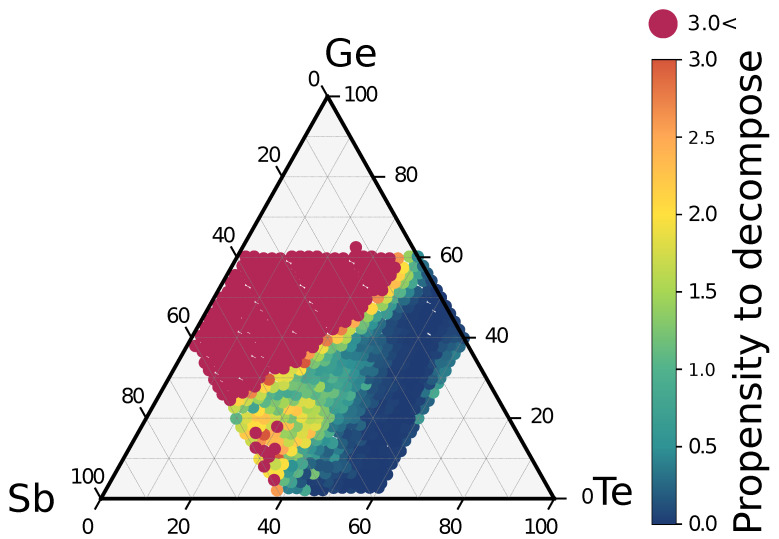
Map of the decomposition propensity of the cubic phase of GST alloys on the ternary phase diagram (see text). Low values (blue region) indicate a low propensity to decompose. The decomposition propensity is computed by counting the exothermic decomposition channels weighted by the modulus of their reaction free energy (in eV/atom).

We then attempted to quantify the decomposition propensity by counting the exothermic decomposition channels weighted by their reaction free energy. In this calculation, we now include all the possible products in the central region of the ternary phase diagram (all the 698 equally spaced alloys) even if they appear in a small atomic fraction in the decomposition product. This is done because a reaction with a low fraction of the GSTX${}^{\prime }$Y${}^{\prime }$Z${}^{\prime }$ products can still be strongly exothermic due to the formation of a large fraction of GeTe. This procedure is repeated now for all possible starting GSTXYZ alloys and we then obtain the map of the propensity to decompose for alloys in the central region of the phase diagram shown in Figure 5. The decomposition propensity is low again along the GeTe-Sb${}_{2}$Te${}_{3}$ tie-line but also along the Sb-GeTe isoelectronic line. This map suggests a possible tuning of the alloy composition to minimize the decomposition propensity by still keeping a higher $T_{X}$, as we discuss below.

In principle, it would be desirable to select an alloy with a sufficiently high $T_{X}$ that still switches between the crystalline and amorphous phases in a homogeneous manner with no phase separation. This would allow the minimization of the cell-to-cell variability and the reduction of the drift of the set state that could arise from the presence of a partially crystallized system or of nanocrystallites with different compositions that may undergo coarsening. The phase separation itself is believed to be the origin of the raise of $T_{X}$ because of the mass transport on a long length scale involved in phase segregation [22]. However, previous analysis of DFT models of the amorphous phase as a function of composition revealed that moving away from the GeTe-Sb${}_{2}$Te${}_{3}$ tie-line by increasing either the Ge or Sb content along the Ge-GST124 [27], Sb-GeTe [56] and Ge-Sb${}_{2}$Te${}_{3}$ lines [42], leads to an amorphous network which is more and more dissimilar from the cubic crystal. In the rocksalt phase, all atoms are in an octahedral bonding geometry with no homopolar bonds. On the contrary, in the amorphous phase, a fraction of Ge atoms are in a tetrahedral bonding geometry favored by the presence of Ge–Ge bonds [54]. The fraction of Ge–Ge bonds and of tetrahedra increases by increasing the Ge content but also the Sb fraction because of the lack of a sufficient amount of Te to form Ge–Te bonds [27,56]. Another indicator of the bonding topology in the amorphous phase is given by the distribution of rings, that is, the shortest closed loops among atoms connected by bonds. In GST225 and GeTe, the ring distribution is dominated by four-membered ABAB rings [58,59] (A = Ge/Sb, B = Te), which are the building blocks of the cubic rocksalt crystal. The four-membered rings are present in the amorphous phase but also in the supercooled liquid phase above *T*${}_{g}$ where crystallization takes place [59]. Molecular dynamics simulations of the crystallization process [59,60,61] have shown that the reorientation of the four-membered rings is the key process in the formation of cubic crystalline nuclei. By increasing the Ge and Sb content at the expense of Te, the fraction of four-membered rings reduces while the number of five- and six-membered rings increases [27,42,56]. Homopolar bonds, tetrahedra and longer rings in place of four-membered ones are all structural features hindering the crystallization since they are not present in the crystal [3,27,42,56]. Therefore, we might conceive to reduce the crystal nucleation rate and then improve data retention by increasing the Ge or Sb fraction to enhance the dissimilarity between the crystal and amorphous phases by moving away from the GeTe-Sb${}_{2}$Te${}_{3}$ pseudobinary line. The propensity map in Figure 5 tells us that if we increase the Ge or Sb content by keeping close to the Sb-GeTe line we can also minimize the propensity to decompose during crystallization. It remains to be seen if this strategy is suitable to raise $T_{X}$ sufficiently for applications in embedded memories.

**Figure 6:**
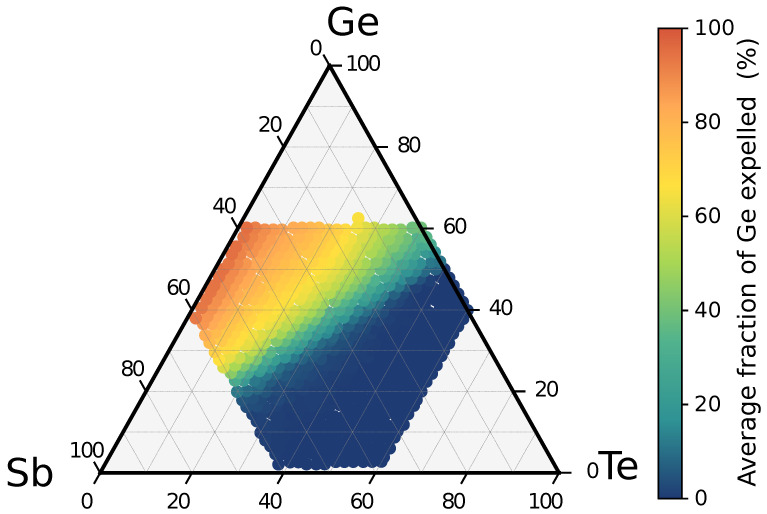
Map of the average fraction of crystalline Ge in atomic % segregated in the exothermic decomposition reactions of GST alloys in the central part of the ternary phase diagram (see text for definition).

As a final result, we report in Figure 6 a map of the average atomic fraction of segregated Ge along the decomposition pathways of a generic GSTXYZ alloy in the ternary phase diagram. This average fraction is computed by summing the atomic fraction of Ge (in the crystalline form) formed in each exothermic decomposition path weighted by its reaction free energy Δ as $Ge_{av}\left(GSTXYZ\right)=\frac{\sum_{i}c_{i}\left|\Delta_{i}\right|}{X\sum_{i}\left|\Delta_{i}\right|}$, where the sum runs over the decomposition channels and $c_{i}$ is the fraction of Ge formed in reaction *i* (see Equation (1)). As expected, there is a rough correspondence between the average fraction of segregated Ge (Figure 6) and the propensity to decompose (Figure 5), but the map of the decomposition propensity shows some finer details (e.g., a lower decomposition propensity along the Sb–GeTe line), which are not present in the map of Figure 6.

## 4. Discussion and Conclusions

The analysis of the decomposition pathways suggests that the crystallization of Ge-rich alloys could lead to the formation of less Ge-rich cubic crystals with different compositions. The coexistence of different competitive decomposition channels might affect the cell-to-cell variability and the drift in the set state of the memory. However, we must remark that the decomposition maps in Figure 3 correspond to the final result of the decomposition reaction with all products in the crystalline state. This process is, however, likely to occur in different steps. For instance, in the real samples, Ge could migrate to enrich the amorphous region surrounding the crystallizing region depleted in Ge. The crystallization of Ge inside the further Ge enriched amorphous region will occur at a later stage or at higher temperature [26].

Therefore, although the decomposition maps for the individual alloy (Figure 3) and the map of the decomposition propensity (Figure 5) suggest possible strategies to mimimize phase segregation, the result of the actual crystallization process is expected to strongly depend on the details of the thermal history of the sample. In fact, it was already noted that the kinetics of crystallization are different for the as-deposited and melt quenched amorphous phase annealed above *T*${}_{g}$, and it is also different from phase separation occurring by directly quenching the liquid below melting [24]. Furthermore, in a nanoscaled device, the switching between the amorphous and the crystalline phases occurs only in a small dome above the heater in the mushroom architecture. The nature of the surrounding material might also affect the expulsion of Ge from the crystallizing region into the outer part of the transforming dome. The forming operation is indeed known to affect the switching process because it controls the composition of both the transforming dome and the surrounding region [18]. The possible formation of a nanocomposite made of crystalline grains of different compositions is also expected to be strongly affected by nanoconfinement; for instance, in a multilayer geometry such as that explored with Sb${}_{2}$Te${}_{3}$/TiTe${}_{2}$ heterostructures for neuromorphic applications in Ref. [62]. We thus simply mention that nanostructuring Ge-rich GST alloys in multilayers might represent another possible strategy for mitigating the phase separation.

In summary, by means of high-throughput DFT calculations we have provided a comprehensive analysis of the possible decomposition pathways that GST amorphous alloys could undergo during crystallization. This analysis focuses in particular on Ge-rich GST alloys of interest for applications in embedded memories for the automotive sector that require high crystallization temperatures.

The calculation of the reaction free energy for the phase separation of crystalline Ge-rich GST into crystalline Ge and less Ge-rich cubic alloys allowed us to identify the thermodynamically more viable decomposition paths. Other kinetic effects related to the process of segregation of Ge might, however, affect the actual products of the phase separation as discussed above. Although these effects cannot be addressed by DFT methods, the recent developments of machine learning interatomic potentials for large scale simulations [63,64,65] (several thousands of atoms for tens of ns), eventually supplemented by accelerating techniques such as the metadynamics method [66,67], pave the way for the direct molecular dynamics simulations of the crystallization of Ge-rich GST alloys to enlighten the intermediate steps of the whole decomposition process.

## Data Availability

The data that support the findings of this study are available from the corresponding author upon reasonable request.

## Abbreviations

The following abbreviations are used in this manuscript:

GST	GeSbTe
PCMs	phase change memories
GSTXYZ	Ge${}_{X}$Sb${}_{Y}$Ye${}_{Z}$
DFT	Density Functional Theory
PBE	Perdew, Burke and Ernzerhof
vdW	van der Waals
BZ	Brillouin zone
SQS	Special quasi-random structure
